# Methylphenidate Use and Infectious Diseases in Children With Attention Deficit and Hyperactivity Disorder: A Population-Based Study

**DOI:** 10.3389/fmed.2021.787745

**Published:** 2022-02-08

**Authors:** Vincent Chin-Hung Chen, Kai-Liang Kao, Yi-Lung Chen, Shu-I Wu, Min-Jing Lee, Michael Gossop

**Affiliations:** ^1^Department of Psychiatry, Chiayi Chang Gung Memorial Hospital, Chiayi, Taiwan; ^2^Department of Psychiatry, Chang Gung University, Taoyuan, Taiwan; ^3^Division of Infectious Diseases, Department of Pediatrics, Far Eastern Memorial Hospital, New Taipei City, Taiwan; ^4^Department of Healthcare Administration, Asia University, Taichung, Taiwan; ^5^Department of Psychology, Asia University, Taichung, Taiwan; ^6^Department of Medicine, Mackay Medical College, New Taipei City, Taiwan; ^7^Department of Psychiatry, Mackay Memorial Hospital, Taipei, Taiwan; ^8^National Addiction Centre, Institute of Psychiatry, King's College London, London, United Kingdom

**Keywords:** attention deficit and hyperactivity disorder, visits to emergency departments, infectious diseases, methylphenidate (MPH), hospitalizations

## Abstract

**Objective:**

Children with attention deficit hyperactivity disorder (ADHD) have more visits to the emergency department (ED) due to injuries than those without ADHD. However, no study has investigated whether children with ADHD have more ED visits or hospitalizations due to infectious diseases (IDs) and whether methylphenidate (MPH) treatment may reduce the risk.

**Method:**

The incidence of ID-related ED visits or hospitalizations was defined as the main outcome. The Cox regression and conditional Poisson regression models were calculated to estimate hazard ratios (*HR*s) in the population level and relative risks for the self-controlled case series design, respectively.

**Results:**

Children with ADHD had higher rates of emergency visits (*HR* = 1.25, 95% *CI*: 1.23~1.27) and hospitalizations (*HR* = 1.28, 95% *CI*: 1.26~1.31) due to IDs than those without ADHD. In the ADHD subgroup, those who received MPH treatment have a reduced risk of emergency visits (*HR* = 0.10, 95% *CI*: 0.09~0.10) and hospitalizations (*HR* = 0.73, 95% *CI*: 0.71~0.75), compared to those without treatment. The risk of ID-related emergency visits decreased to 0.21 (95% *CI*: 0.21~0.22); and hospitalizations decreased to 0.71 (95% *CI*: 0.69~0.73). Within self-controlled analysis, it is demonstrated that compared with non-MPH exposed period, children with ADHD had significantly decreased risks for infection-related emergency visits (*RR* = 0.73, 95% *CI*: 0.68~0.78) or hospitalizations (*RR* = 0.19, 95% *CI*: 0.17~0.21) during MPH-exposed periods.

**Conclusions and Relevance:**

This is the first study that reported an increased risk of ID-related healthcare utilizations in children with ADHD compared to those without, and that such risks may be significantly reduced in ADHD children that received MPH treatment.

## Introduction

Attention deficit and hyperactivity disorder (ADHD) is one of the most common neurodevelopmental disorders in childhood (1). ADHD is characterized by inattentive, hyperactive, or impulsive behavior (2). With a worldwide prevalence of 5~20% (3), children with ADHD have serious occupational or social impairments of poor academic performances, delinquency, substance misuse, or social incompetence (4) that continue through adulthood. In addition, an up to 2-fold risk of increased mortality (5, 6) has been found in children with ADHD compared to the general population. Increased risks of accidents, physical injuries, suicides, or homicides were shown to be related to the excessive mortality (5, 6).

Emerging evidence has supported the treatment effects of methylphenidate (MPH) for ADHD symptoms or related adverse behavioral outcomes (7–9), as well as healthcare utilizations, such as visits to the emergency departments (EDs). Studies showed that MPH may account for 13~34% decrease in risks of fracture (10–13), 34~51% of brain injuries (12, 14), 19% of substance-related events (15), or 19~72% of reductions in suicide (16, 17). An 11% decrease in visits to EDs due to trauma (18), 38~42% of ED visits due to motor vehicle crashes (19), or 58% of transport accidents (20) were found to be associated with MPH treatment.

Reductions in the prevalence of infectious diseases (IDs) in children has contributed to the decrease in childhood mortality in the 20th century (21, 22). Yet, with numbers of 905,059 deaths from lower respiratory tract infections among younger children, and 38,325 deaths from diarrheal diseases among older children, IDs still account for nearly one million annual deaths all over the world (21), and are still the main cause of emergency visits or hospitalizations in children. The main prevention methods for IDs are careful attention on personal hygiene and avoid contacts with pathogens (23). However, recommendations, such as wearing masks, washing hands, or keeping social distances, may be difficult for children with ADHD to follow, particularly among younger children (24). Previous research has described that patients with ADHD had a 3.36-fold higher risk of sexually transmitted infections than those without ADHD (25). In addition, they found that use of ADHD medications may significantly decrease 30~41% of such infections (25). However, no studies have examined whether children with ADHD have higher risks of serious respiratory, gastrointestinal, or urinary tract infections that require visits to EDs or hospitalizations. Additionally, possible effects of MPH treatments in ADHD children with or without relevant neurodevelopmental comorbidities in the risk of IDs-related emergency visits or hospitalizations have never been explored. Investigations on these associations may help the prevention and management of IDs in children with ADHD.

Using a large population-based dataset, we investigated whether higher rates of IDs-related emergency visits or hospitalizations were found in children with ADHD compared to those without. Between-subject comparisons of ADHD children with or without MPH treatment, and within-comparison analysis comparing subsequent risks of IDs-related emergency visits and hospitalizations between MPH-exposed or unexposed periods were performed to elucidate the treatment effects of MPH.

## Methods

### Study Design and Participants

In this retrospective cohort study, we used data from the Taiwan National Health Insurance Research Database (NHIRD) under the aegis of the National Health Research Institute, which includes data on outpatient, ambulatory, and hospital inpatient care. Taiwan launched a single-payer National Health Insurance (NHI) program on March 1, 1995. The NHI covers the delivery of all healthcare services to over 99.5% of the national population (26). To avoid inappropriate claims outside the indications, all treatment claims are scrutinized by the NHI Review Committee on regular basis (every 3 months) to inspect the appropriateness of disease indications and treatment claims. Clinicians that made insurance claims not meeting the prescription criteria would be fined and deducted 10 times the claim value. The research database of NHIRD contains information of patients' demographic data, the medical institution visited, diagnostic codes, the drugs prescribed, the date of any prescriptions given, and any claimed medical expenses. The database has been used in many epidemiologic studies in Taiwan (10, 27). Several validation studies have shown that the dataset represents moderate to high sensitivity and positive predictive values (28, 29).

### Exposure Assessment

The ADHD cohort was selected from NHIRD. We selected individuals born between 1997 and 2005 because we applied data from NHIRD from 1997 to 2013 and the age counterparts without ADHD were selected from the Longitudinal Health Insurance Database 2005 (LHID). LHID 2005 contains data for 1,000,000 enrollees randomly sampled from the 2005, thus enrollees in the LHID 2005 would not be born after 2005. The distribution of sex and age of the sampled enrollees in the LHID 2005 did not differ significantly from that of the general population (29). We identified 75,141 patients with ADHD who received at least one inpatient diagnosis of ADHD (International Classification of Disease, 9th revision [ICD-9] code: 314) or more than two outpatient diagnoses within 1 year between 1997 and 2013. For non-ADHD group, after the removal of patients with ADHD, 94,567 participants from the LHID2005 remained. This study was reviewed and approved by the Institutional Review Board of Chang Gung Memorial Hospital, Taoyuan City, Taiwan.

### Outcomes

The main outcome of this study was IDs-related visits of the ED or hospitalizations. Participants were followed-up for the incidence of IDs-related ED visits or hospitalizations as an outcome, or until the end of 2013. IDs-related diagnoses pediatricians in Taiwan often use indicating the clinical infectious conditions that required visits to the ED or hospitalizations included: meningitis (ICD-9-CM codes: 320.xx~323.xx), conjunctivitis (372.xx), acute otitis media (382.xx), upper respiratory infections, pharyngitis, laryngitis, bronchitis (461.xx ~ 466.xx), pneumonia (480.xx ~ 490.xx), gastritis, duodenitis (535.xx), urinary tract infection (599.xx), fever (780.6), and abdominal pain (789.xx). In addition, we reported the top three most frequently primary diagnosis for IDs-related ED visits and hospitalizations.

### Treatment Status of MPH

We ascertained the MPH treatment status by dispensed prescriptions recorded in the prescribed drug register. MPH (ATC codes: N06BA04; short-acting or extended release) was the only stimulant approved for treating ADHD in Taiwan, and was regarded as the first-line treatment for ADHD by the Taiwan National Insurance. Indications approved in the reimbursement system for prescribing short-acting MPH HCL are ADHDs and narcolepsy; and could be prescribed by doctors of all specialty in Taiwan. Further criteria for prescribing MPH HCL Extended Release were limited to patients between 6 and 18 years old diagnosed with ADHD according to DSM or ICD criteria, and those who cannot tolerate the side effects or gained beneficial effects from short acting MPH HCL (Ritalin). For those who meet the requirements, have been treated with MPH, and still need to take the medication after the age of 18, their medical history and reasons for use must be recorded in detail in their medical records to be reimbursed. Since 2017, atomoxetine (ATX), a non-stimulant, received approval for ADHD treatment in Taiwan. Both medications were approved for patients with age of 6 or older. However, compared with MPH, there is a much lower rate of prescription of ATX (4% in all patients with ADHD) (30). ATX is only recommended for cases with unsatisfied treatment outcomes (i.e., inefficacy and intolerability) from MPH in Taiwan. Thus, it can be assumed that those patients who received ATX have had prior MPH treatment exposure. Therefore, we included patients with MPH exposure only in this study. We investigated the treatment duration effect by examining the MPH use in the ADHD cohort.

### Assessment of Other Characteristics

Several covariates were selected, such as sex, age, and psychiatric comorbidity comprising autism spectrum disorder (ASD; ICD-9 code: 299), oppositional defiant disorder (ODD; ICD-9 code: 313.81) or conduct disorder (CD; ICD-9 code: 312), major depressive disorder (MDD; ICD-9 codes: 296.2–296.23, 311, and 300.4), tic disorders (ICD-9 code: 307.2), epilepsy (ICD-9 code: 345), and intellectual disabilities (ICD-9 codes: 317–319) at any time during the study period.

### Statistical Analysis

All data management and statistical analyses were performed using SAS Version 9.4 (SAS Institute Inc., Cary, NC, USA) and R 4.0.2 (R Foundation for Statistical Computing, Vienna, Austria). To describe the distribution of the study population, a chi-square (χ^2^) test was used to compare the characteristics between the ADHD and control groups. This study performed two different analyses: between subject comparison and within subject comparison. We used the one-to-one propensity score matching to draw a comparison cohort by exact matching for gender and greedy matching for age group and comorbidity. We matched subjects on the logit of the propensity score using a caliper of a width of 0.001. Standardized mean differences were used to evaluate the difference of matching variables between the ADHD and non-ADHD groups, and the MPH user and non-MPH user groups among ADHD. A standardized mean difference of 0.2 or greater indicates a notable difference between the two groups (31).

For between-subject comparison, the adjusted risk of emergency visits or hospitalizations between those with ADHD and without was estimated using a robust Cox proportional hazard model to take the propensity score matching strata into account. The results are presented as adjusted hazard ratios (aHRs) with 95% *CI*s. A similar analysis was conducted between ADHD with or without MPH medication. For categorization of MPH medication, if individuals with ADHD took their first MPH medication after their emergency visits or hospitalizations, they were categorized as non-MPH medication. For within-self comparisons, we used the self-controlled case series (SCCS) model, in which time was divided into periods similar to the conditional Poisson regression model, with each patient as a separate stratum (i.e., the patient served as his or her own control). Individual's record of MPH medication was used from the entire study period (by the end of 2013). The results are presented as relative incidences (*RR*s) with 95% *CI*s. The RR estimated from the SCCS model indicates the risk of emergency visits or hospitalizations during the period an individual took MPH compared with the period when they did not take MPH. The SCCS model automatically adjusts for all time-invariant factors (e.g., sex) for the same patient before and during the follow-up, and we further adjusted for time-varying variables (i.e., age).

The effect of exposure to MPH was defined as 3 months after the completion of each MPH treatment, and the length of effect period for MPH was based on the suggestion of previous studies (32). We further examined whether the effect remained in different months from 1- to 3-month. Thus, the effect period was split into three 1-month effect periods: 0~30, 31~60, and 61~90 days from the end of each treatment period. Finally, we combined the three effect periods into one to report the average pooled estimate of MPH use for IDs-related emergency visits or hospitalizations. Supplementary analyses regarding comparisons of risks between the subtypes of inattentive or hyperactivity and short-acting or long-acting MPH were also performed.

## Results

**Figure 1:**
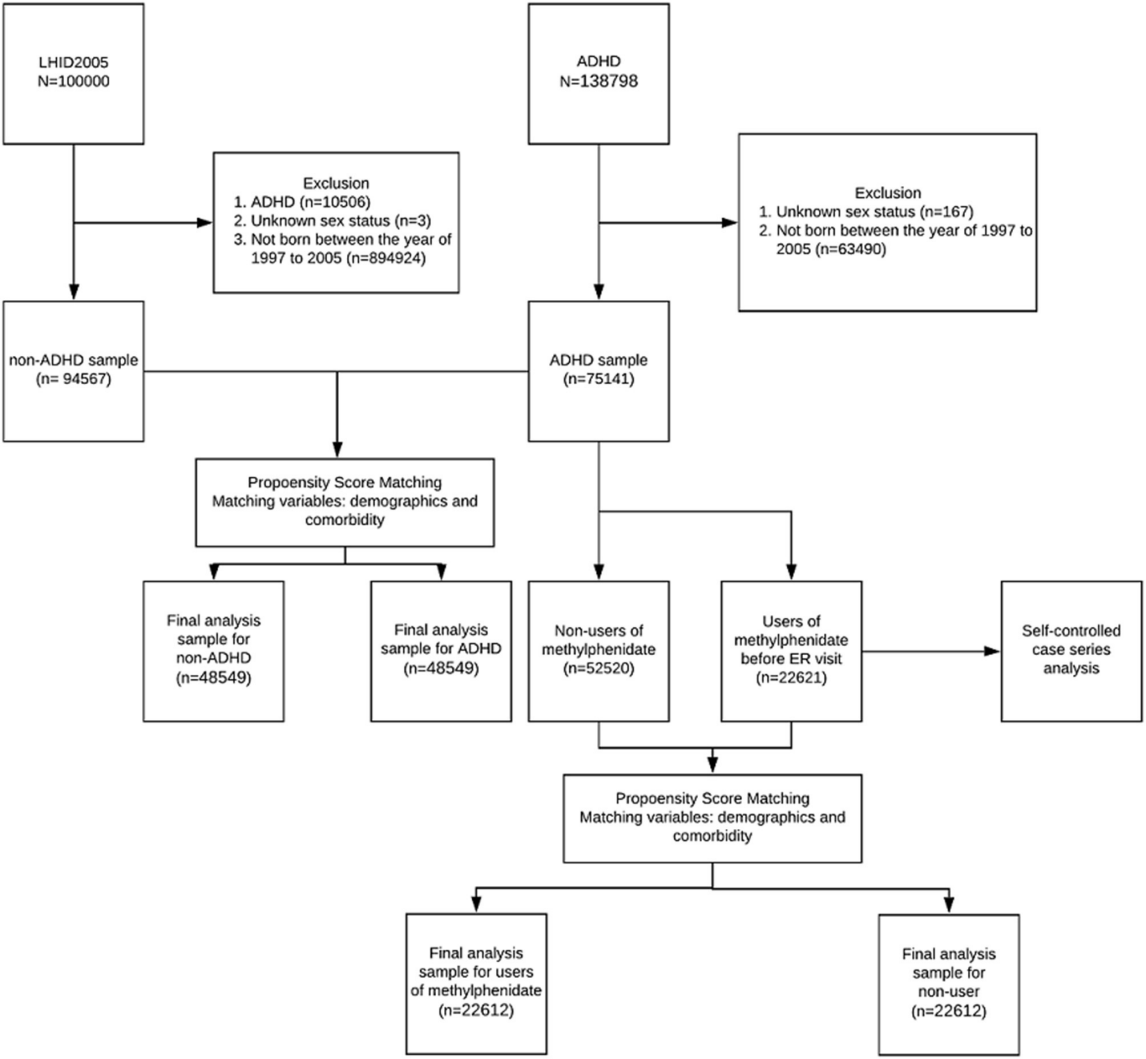
The flow chart for selections, propensity score-matching, and self controlled case series comparisons among our study subjects.

Figure 1 illustrates the methodology, case selection, and comparison method used, with numbers of people in each group. Table 1 shows the characteristics of children with and without ADHD. After propensity score matching, there were no significant differences in gender, age groups, or comorbidities between the ADHD and non-ADHD groups and standardized mean difference (all standardized mean differences <0.2). The three most common IDs that required visits to the ED or hospitalizations for patients with ADHD were (1) acute upper airway respiratory infections, (2) fever, (3) acute bronchiolitis; or (1) bronchopneumonia, (2) acute bronchiolitis, and (3) pneumonia, respectively.

**Table 1:** Demographic characteristics of ADHD and non-ADHD youths after propensity score matching.

	**ADHD**	**Non-ADHD**	**Standardized mean difference**
**Characteristics**	***n* = 48,549**	***n* = 48,549**	
Sex, *n* (%)
Female	10,260 (21.13)	10,260 (21.13)	<0.001
Male	38,289 (78.87)	38,289 (78.87)	
Age group
Age ≤ 12	21,129 (43.52)	21,111 (43.48)	−0.147
Age >12	27,420 (56.48)	27,438 (56.52)	
Subtype of ADHD
Inattention	21,538 (44.4)	-	-
Hyperactivity	35,681 (73.5)	-	-
Autism spectrum disorder, *n* (%)	304 (0.63)	309 (0.64)	
Tic disorders, *n* (%)	846 (1.74)	843 (1.74)	<0.001
Epilepsy, *n* (%)	1,590 (3.28)	15,93 (3.28)	<0.001
ODD/CD, *n* (%)	186 (0.38)	184 (0.38)	<0.001
Major depressive disorder, *n* (%)	78 (0.16)	88 (0.18)	0.002
Intellectual disabilities, *n* (%)	710 (1.46)	697 (1.44)	−0.001
Total emergency room visit, *n* (%)	34,411 (70.88)	31,530 (64.94)	-
Infectious diseases-related emergency visits	16,962 (34.94)	14,471 (29.81)	-
Top three diagnoses of infectious diseases-related emergency visits		-
1. Acute upper respiratory infections of unspecified site (ICD-9-CM: 465.9)	3,077 (6.34)	2,456 (5.06)	-
2. Fever (ICD-9-CM: 780.6)	2,657 (5.47)	2,710 (5.58)	-
3. Acute bronchitis (ICD-9-CM: 466.0)	1,506 (3.10)	1,266 (2.61)	-
Total hospitalizations, *n* (%)	3,2013 (65.94)	27,202 (56.03)	
Infectious diseases-related hospitalizations	21,462 (44.21)	17,611 (36.27)	-
Top three diagnoses of infectious diseases-related hospitalizations in ADHD children	-
1. Bronchopneumonia (ICD-9-CM: 485)	3,644 (15.67)	3,142 (16.38)	-
2. Acute bronchiolitis (ICD-9-CM: 4661)	2,319 (9.97)	1,700 (8.86)	
3. Pneumonia (ICD-9-CM: 486)	2,221 (9.55)	1,895 (9.88)	-
Median age of first hospitalization (IQR), years	7.11 (1.27~13.21)	3.98 (0.82~12.38)	-
Median age of first emergency room visit (IQR), years	5.28 (2.22~11.54)	7.94 (2.97~13.04)	-

*ADHD, attention deficit hyperactivity disorder; CD, conduct disorder; IQR, interquartile range; ODD, oppositional defiant disorder*.

After propensity score matching, there were no significant differences in gender, age groups, and comorbidities between the MPH user and non-user subgroups. Table 2 summarized the results of incidences for emergency visits or hospitalizations between the ADHD and non-ADHD groups using the propensity score matched Cox proportional hazard regression model. Compared with the non-ADHD group, the ADHD group had higher relative incidences of overall emergency visits (a*HR* = 1.25, 95% *CI*: 1.23~1.27), IDs-related emergency visits (a*HR* = 1.35, 95% *CI*: 1.32~1.38), all hospitalizations (a*HR* = 1.28 (1.26~1.31), and IDs-related hospitalizations (a*HR* = 1.28, 95% *CI*: 1.25~1.30) after controlling for demographics and psychiatric comorbidities using propensity score matching. Results from our subanalyses differentiating subtypes of inattentive or hyperactivity revealed similar patterns as our main analyses (Supplementary Table 1). These differences were robust in different demographic subgroups between the ADHD and non-ADHD groups but were less consistent in different strata of psychiatric comorbidities. Significant differences between ADHD and non-ADHD groups were only observed in the strata of epilepsy for all or IDs-related emergency visits; and in the strata of intellectual disability for all or IDs-related hospitalizations.

**Table 2:** Cox proportional hazard regression analysis with propensity score matching for risks of infectious diseases-related emergency room visits and hospitalizations between ADHD and non- ADHD youths stratified by demographic variables and comorbidities.

	**All emergency visits**		**Infectious diseases-related emergency visits**		**All hospitalizations**		**Infectious diseases-related hospitalizations**	
**Variable**	**aHR (95% CI)**	***p***	**aHR (95% CI)**	***p***	**aHR (95% CI)**	***p***	**aHR (95% CI)**	***p***
ADHD	1.25 (1.23~1.27)	<0.001	1.35 (1.32~1.38)	<0.001	1.28 (1.26~1.31)	<0.001	1.28 (1.25~1.30)	<0.001
Male sex (reference = non-ADHD)	1.25 (1.23~1.27)	<0.001	1.35 (1.32~1.39)	<0.001	1.27 (1.24~1.29)	<0.001	1.26 (1.23~1.29)	<0.001
Female sex (reference = non-ADHD)	1.25 (1.21~1.30)	<0.001	1.35 (1.29~1.42)	<0.001	1.36 (1.31~1.41)	<0.001	1.37 (1.31~1.43)	<0.001
Age ≤ 12 (reference = non-ADHD)	1.25 (1.22~1.28)	<0.001	1.33 (1.29~1.37)	<0.001	1.29 (1.26~1.32)	<0.001	1.27 (1.23~1.31)	<0.001
Age > 12 (reference = non-ADHD)	1.28 (1.25~1.31)	<0.001	1.41 (1.36~1.45)	<0.001	1.28 (1.25~1.31)	<0.001	1.28 (1.25~1.32)	<0.001
Autism spectrum disorder (reference = non-ADHD)	1.05 (0.87~1.27)	0.589	1.10 (0.84~1.44)	0.468	0.91 (0.75~1.11)	0.363	0.99 (0.78~1.27)	0.941
Tic disorders (reference = non-ADHD)	0.98 (0.87~1.09)	0.675	1.08 (0.92~1.26)	0.364	1.01 (0.90~1.14)	0.853	1.00 (0.87~1.15)	0.976
Epilepsy (reference = non-ADHD)	1.17 (1.08~1.26)	<0.001	1.28 (1.13~1.43)	<0.001	0.96 (0.88~1.04)	0.281	0.99 (0.90~1.08)	0.766
ODD/CD (reference = non-ADHD)	1.17 (0.91~1.49)	0.218	1.14 (0.79~1.64)	0.480	1.19 (0.91~1.54)	0.199	1.15 (0.85~1.56)	0.361
Major depressive disorder (reference = non-ADHD)	1.11 (0.78~1.58)	0.558	1.06 (0.62~1.81)	0.833	1.14 (0.78~1.68)	0.492	0.78 (0.50~1.22)	0.283
Intellectual disabilities (reference = non-ADHD)	1.02 (0.89~1.15)	0.818	1.09 (0.91~1.30)	0.346	0.82 (0.72~0.93)	0.002	0.83 (0.72~0.95)	0.009

*ADHD, attention deficit hyperactivity disorder; CD, conduct disorder; ODD, oppositional defiant disorder. Adjusted cox proportional hazard regression analysis with propensity score matching was conducted adjusted for variable list in Table 1*.

Table 3 shows the influences of the MPH cumulative effect on emergency visits or hospitalizations in the subgroup of children with ADHD. For the whole sample, compared to patients with ADHD not taking MPH, those taking MPH for <90 days had an adjusted *HR* of 0.21 (95% *CI*: 0.21~0.22) for IDs-related emergency visits; and 0.71 (95% *CI*: 0.69~0.73) for IDs-related hospitalizations. Those taking MPH for ≥90 days had an a*HR* of 0.12 (95% *CI* 0.09~0.16) for IDs-related emergency visits and 0.72 (95% *CI*: 0.63~0.83) for IDs-related hospitalizations. The results for the trend test were significant (*p* < 0.01). In addition, all subgroup analyses of different demographics or comorbidities revealed similar patterns (Table 3).

**Table 3:** Cox proportional hazard regression model analysis with the use of methylphenidate on hospitalizations or emergency room visits in ADHD youth stratified by demographic variables and comorbidities.

	**All emergency visits**		**Infectious diseases-related emergency visits**		**All hospitalizations**		**Infectious diseases-related hospitalizations**	
**Variable**	**HR (95% CI)**	***p***	**HR (95% CI)**	***p***	**HR (95% CI)**	***p***	**HR (95% CI)**	***p***
Use of methylphenidate (reference = non-users)	1.00	-	1.00	-	1.00	-	1.00	-
<90 DDD	0.10 (0.09~0.10)	<0.001	0.21 (0.21~0.22)	<0.001	0.73 (0.71~0.75)	<0.001	0.71 (0.69~0.73)	<0.001
≥90 DDD	0.01 (0.01~0.02)	<0.001	0.12 (0.09~0.16)	<0.001	0.79 (0.71~0.89)	<0.001	0.72 (0.63~0.83)	<0.001
Male sex (reference = non-users)	0.09 (0.09~0.09)	<0.001	0.21 (0.20~0.22)	<0.001	0.74 (0.72~0.76)	<0.001	0.72 (0.70~0.74)	<0.001
Female sex (reference = non-users)	0.10 (0.09~0.11)	<0.001	0.22 (0.20~0.24)	<0.001	0.69 (0.65~0.72)	<0.001	0.67 (0.63~0.71)	<0.001
Younger age (reference = non-users)	0.05 (0.05~0.06)	<0.001	0.15 (0.14~0.16)	<0.001	0.66 (0.63~0.69)	<0.001	0.65 (0.62~0.69)	<0.001
Older age (reference = non-users)	0.11 (0.11~0.11)	<0.001	0.21 (0.20~0.22)	<0.001	0.76 (0.74~0.78)	<0.001	0.73 (0.71~0.76)	<0.001
Autism spectrum disorder (reference = non-users)	0.14 (0.13~0.15)	<0.001	0.27 (0.24~0.30)	<0.001	0.74 (0.69~0.79)	<0.001	0.71 (0.66~0.77)	<0.001
Tic disorders (reference = non-users)	0.10 (0.09~0.12)	<0.001	0.21 (0.17~0.24)	<0.001	0.81 (0.74~0.89)	<0.001	0.80 (0.71~0.89)	<0.001
Epilepsy (reference = non-users)	0.13 (0.11~0.14)	<0.001	0.23 (0.19~0.27)	<0.001	0.79 (0.72~0.86)	<0.001	0.78 (0.71~0.86)	<0.001
ODD/CD (reference = non-users)	0.09 (0.08~0.10)	<0.001	0.19 (0.17~0.22)	<0.001	0.73 (0.68~0.79)	<0.001	0.71 (0.65~0.78)	<0.001
Major depressive disorder (reference = non-users)	0.13 (0.10~0.15)	<0.001	0.18 (0.14~0.25)	<0.001	0.69 (0.58~0.82)	<0.001	0.55 (0.45~0.67)	<0.001
Intellectual disabilities (reference = non-users)	0.13 (0.12~0.13)	<0.001	0.27 (0.24~0.29)	<0.001	0.70 0.66~0.74)	<0.001	0.67 (0.63~0.72)	<0.001

*CD, conduct disorder; DDD, Defined Daily Dose; ODD, oppositional defiant disorder. Twenty-two thousand forty-six users of methylphenidate with their cumulative DDD smaller than 90 and 566 users of methylphenidate with their cumulative DDD equal to or >90*.

Table 4 presents the within-self comparisons comparing MPH exposed period and non-exposed periods in patients with ADHD using the SCCS model with the adjustment for age effect. Within-patient comparisons revealed a significant reduction in the incidence of IDs-related emergency visits in effect periods from 0 to 30 (*RR*: 0.78, 95% *CI*: 0.71~0.84), 30 to 60 (*RR*: 0.69, 95% *CI*: 0.61~0.79), and 60 to 90 days (*RR*: 0.64, 95% *CI*: 0.54~0.76). An average pooled estimate of these effect periods indicated an *RR* of 0.73(95% *CI*: 0.68~0.78). Significant reductions in the incidence of IDs-related hospitalizations were observed with an average pooled estimate of these effect periods at an *RR* of 0.19 (95% *CI*: 0.17~0.21).

**Table 4:** Conditional Poisson regression model for self-controlled case series study design of hospitalizations and emergency room visits in ADHD youths with use of methylphenidate and emergency room visit.

	**All emergency visit**			**Infectious diseases-related emergency visits**			**All hospitalizations**			**Infectious diseases-related hospitalizations**		
	***N*** **=** **20,754**			***N*** **=** **10,256**			***N*** **=** **20,933**			***N*** **=** **13,814**		
**Variable**	**RR^a^**	**95% CI**	***p***	**RR**	**95% CI**	***p***	**RR** ^a^	**95% CI**	***p***	**RR^a^**	**95% CI**	***p***
Exposures of methylphenidate										
0~30 days	0.39	(0.26~0.36)	<0.001	0.78	(0.71~0.84)	<0.001	0.19	(0.17~0.21)	<0.001	0.19	(0.17~0.22)	<0.001
30~60 days	0.34	(0.32~0.37)	<0.001	0.69	(0.61~0.79)	<0.001	0.19	(0.17~0.22)	<0.001	0.19	(0.16~0.23)	<0.001
60~90 days	0.36	(0.32~0.39)	<0.001	0.64	(0.54~0.76)	<0.001	0.19	(0.16~0.23)	<0.001	0.21	(0.14~0.23)	<0.001
0~90 days	0.36	(0.35~0.38)	<0.001	0.73	(0.68~0.78)	<0.001	0.18	(0.14~0.23)	<0.001	0.19	(0.17~0.21)	<0.001

a *Relative incidence (RR) was calculated by conditional Poisson regression, adjusted for all time-invariant covariates that are constant within each individual during the follow-up and time-varying covariate (i.e., age stage)*.

Table 5 presents the analyses of SCCS model in different subgroups. Significant reductions in incidences of IDs-related emergency visits or hospitalizations were found in almost all the demographic and psychiatric comorbidity subgroups, except in IDs-related emergency visits among patients with ADHD comorbid with tic disorder, epilepsy, or major depressive disorder during MPH exposure compared with non-exposed periods.

**Table 5:** SCCS model use of methylphenidate of on hospitalizations and emergency room visits in ADHD youth stratified by demographic variables and comorbidities.

	**All emergency visits**		**Infectious diseases-related emergency visits**		**All hospitalizations**		**Infectious diseases-related hospitalizations**	
**Variable**	**^a^RR (95% CI)**	***p***	**^a^RR (95% CI)**	***p***	**^a^RR (95% CI)**	***p***	**^a^RR (95% CI)**	***p***
Male sex (reference = non-exposed periods)	0.35 (0.34~0.37)	<0.001	0.68 (0.63~0.73)	<0.001	0.18 (0.16~0.20)	<0.001	0.18 (0.16~0.20)	<0.001
Female sex (reference = non-exposed periods)	0.44 (0.40~0.49)	<0.001	1.03 (0.88~1.21)	0.704	0.24 (0.19~0.30)	<0.001	0.24 (0.19~0.30)	<0.001
^b^Younger age (reference = non-exposed periods)	0.44 (0.42~0.47)	<0.001	0.39 (0.32~0.47)	<0.001	0.13 (0.10~0.18)	<0.001	0.13 (0.10~0.18)	<0.001
^b^Older age (reference = non-exposed periods)	0.11 (0.10~0.13)	<0.001	0.75 (0.69~0.81)	<0.001	0.18 (0.16~0.20)	<0.001	0.18 (0.16~0.20)	<0.001
Autism spectrum disorder (reference = non-exposed periods)	0.38 (0.34~0.43)	<0.001	0.80 (0.65~0.97)	0.0226	0.23 (0.18~0.29)	<0.001	0.23 (0.18~0.29)	<0.001
Tic disorders (reference = non-exposed periods)	0.41 (0.35~0.48)	<0.001	0.78 (0.60~1.02)	0.0661	0.17 (0.12~0.26)	<0.001	0.17 (0.12~0.26)	<0.001
Epilepsy (reference = non-exposed periods)	0.43 (0.37~0.49)	<0.001	0.92 (0.72~1.17)	0.486	0.14 (0.10~0.21)	<0.001	0.14 (0.10~0.21)	<0.001
ODD/CD (reference = non-exposed periods)	0.41 (0.36~0.46)	<0.001	0.60 (0.48~0.75)	<0.001	0.16 (0.12~0.23)	<0.001	0.16 (0.12~0.23)	<0.001
Major depressive disorder (reference = non-exposed periods)	0.63 (0.49~0.80)	<0.001	0.85 (0.56~1.27)	0.421	0.39 (0.23~0.66)	<0.001	0.39 (0.23~0.66)	0.003
Intellectual disabilities (reference = non-exposed periods)	0.42 (0.38~0.46)	<0.001	0.68 (0.58~0.80)	<0.001	0.22 (0.17~0.27)	<0.001	0.22 (0.17~0.27)	<0.001

*CD, conduct disorder; DDD, Defined Daily Dose; ODD, oppositional defiant disorder*.

a *Relative incidence (RR) was calculated by conditional Poisson regression, adjusted for all time-invariant covariates that are constant within each individual during the follow-up and time-varying covariate (i.e., age stage)*.

b *Analysis did not adjust for time-varying covariate (i.e., age)*.

We have performed additional analyses comparing results separating the short- and long-acting MPH subgroups in Supplementary Tables 2 and 3. We found that long-acting MPH use was associated with significantly lower risks of all emergency- or IDs-related visits compared with that of short-acting MPH (Supplementary Table 2). Supplementary Table 3 compared effects of short- and long-acting MPH according to different exposure periods using the SCCS model. We found that during the period of long-acting MPH use, lower risks of all emergency visits were lower compared with the period exposed to short-acting MPH in exposure periods of 0~30 or 0~90 days.

## Discussion

This is the first population-based study to report that children with ADHD had increased risks of IDs, and that there is an overall consistent pattern of MPH use and decreased likelihood of IDs among children with ADHD. In addition, using the self-controlled case series analysis, we found that risks of IDs-related emergency visits and hospitalizations decreased 36~81% in the MPH exposed periods compared with the non-exposed periods after adjusting for time-invariant covariates.

Our finding that children with ADHD had higher risks of subsequent IDs than children without ADHD was similar to a recent cohort study reporting significantly increased rates of sexually transmitted infections (STI) in children with ADHD (25). In line with the comments of Chen on possible mechanisms for increased STI in ADHD, that patients might be lacking in safety behaviors, we believe that the increased infection rates in our study subjects maybe due to inadequate personal hygiene or insufficient personal protections (33). From a public health perspective, recommendations, such as wash hands often with soap and water, do not touch eyes, nose, or mouth unless washed hands first, keep the environment clean, cover mouth when coughing, or avoid people that have cold or flu, are extremely relevant for this vulnerable group (33).

Previous research examining the effect of MPH on the risk of IDs-related healthcare utilizations among children with ADHD is lacking. Man et al. described the protective effects of MPH on trauma-related emergency admissions and not on non-trauma ones. In our study, significant reductions in emergency visits or hospitalizations due to upper or lower respiratory or gastrointestinal infections were found in the MPH treated subgroup or exposed periods. Our findings are in agreement with the previous research that reported reduced risks of subsequent STI under short-term or long-term use of ADHD medications in children with ADHD (25). In addition, we found that numbers of IDs-related emergency visits or hospitalizations were significantly reduced regardless of short- or long-term MPH use. A decreased gradient in the risk of emergency visits due to IDs was noticed with the longer-term use of MPH. Our findings indicated that MPH medication treatments for ADHD may be important in preventing subsequent IDs. It is possible that, with MPH treatment, children with ADHD were able to concentrate and learn more thoroughly and effectively about the timing, indications, or correct cleaning methods for hands or respiratory hygiene. Similarly, it is possible that increased attention achieved by MPH treatment may enhance ability or compliance of these children to use personal protective gears (such as gloves or masks) correctly, or implement sterilization or disinfection steps more appropriately to protect them from IDs (34).

Risks of IDs-related hospitalizations from the within-subject analysis did not show a decreased pattern in gradient as the length of exposure to MPH increased. Reasons may be the low overall long-term treatment rates (only 16% of children with ADHD received long-term medication treatments) (25), or the poor compliance to long-term medications that made the impacts of medications not as good in long-term treatment than short-term exposures. The probable reason might partly be due to the possibility that even longer use of MPH would not add further benefits than short-term use when the condition of infection is at a more severe level that requires hospitalization. We found that in patients with ADHD comorbid with tic disorder, epilepsy, or major depressive disorder, exposures to MPH treatment did not add further benefit in preventing IDs-related emergency visits, but could still prevent relevant hospitalizations. However, it can be inferred from the results that MPH exposure could significantly reduce the risk of all emergency visits, that MPH treatments may still enhance the general health status of these subgroups, reduce the occurrences of other causes leading to emergency visits, and still decrease more severe infections that requires hospitalizations.

## Strengths and Limitations

Strengths of this study include, first, the use of a long-term population-based dataset with records of diagnoses and health utilizations that provide detailed information without recall bias. A sample size large enough for sufficient statistical power is an advantage. Second, the within-subject analysis with SCCS was able to help control for the unmeasured time-invariant confounders. Key limitations include, first, diagnoses were obtained from a database established for administrative rather than research purposes. Although this may reflect a naturalistic clinical setting, the underdiagnosis of ADHD or other psychiatric comorbidities might still occur. Second, as for estimating medication exposures, we only have information of prescriptions. There might still be a gap we were not able to identify between medication prescribed and doses that patients actually consumed. Third, although the SCCS design has helped controlled for unmeasured covariates that is constant overtime, there are still time-variant confounders, such as body weight, nutrition status, or non-pharmacological treatments, we were not able to control for. Another possible confounder was the proficiency of parenting skills, such as parents' own standards for cleanliness, protection of health conditions of children, or supervision and correction of hygiene of children, may also affect whether children may be infected. Although this aspect might be seen as a time-invariant confounder, and might be controlled by our SCCS model; with more sufficient information, such as explorations of parenting skills or parental ID conditions within the same period of the infection of child, it may be possible to assess how parenting skills may affect the risk of infection in the child. Last, our results were analyzed from an Asian population with a healthcare system of near-universal coverage, generalizations to other nations may be limited.

## Conclusion

Results of our study suggested that MPH treatment was able to significantly decrease IDs-related emergency visits or hospitalizations, either compared with non-MPH treated children with ADHD, or MPH unexposed periods within the same study subject. Future research on possible pathways of MPH and its protective effects on infections in daily environment of children with ADHD might be explored. Psychoeducation and provisions of early and appropriate MPH treatment are not only important for the traditional academic concerns but might also be helpful in preventing the infection-related premature mortality in this vulnerable population.

## Data Availability Statement

Datasets being analyzed, results being generated and reported in this article can be obtained from the National Health Research Institute of the Ministry of Health and Welfare in Taiwan. Restrictions applied to these data, which were used under license for our study, and so are not publicly available for duplication. Data can be requested only from the Ministry of Health and Welfare.

## Author Contributions

VC, S-IW, Y-LC, and K-LK designed the study and wrote the protocol. Y-LC undertook the statistical analysis. All authors contributed to the writing and have approved the final manuscript.

## Funding

This study was supported by grants from the Chiayi Chang Gung Memorial Hospital, Chiayi, Taiwan (Grant Number: CMRPG6E0271). S-IW is part-funded by the Department of Medical Research, Mackay Memorial Hospital (MMH-109112, MMH-10914, MMH-108121, MMH-108146, MMH-TT-10804, and MMH-TH-10804).

## Conflict of Interest

The authors declare that the research was conducted in the absence of any commercial or financial relationships that could be construed as a potential conflict of interest.

## Publisher's Note

All claims expressed in this article are solely those of the authors and do not necessarily represent those of their affiliated organizations, or those of the publisher, the editors and the reviewers. Any product that may be evaluated in this article, or claim that may be made by its manufacturer, is not guaranteed or endorsed by the publisher.
