# Supplementary material for: Methylphenidate Use and Infectious Diseases in Children With Attention Deficit and Hyperactivity Disorder: A Population-Based Study
Source: Front Med (Lausanne). 2022 Feb 8;8:787745. doi: 10.3389/fmed.2021.787745 (PMC8861374; doi:10.3389/fmed.2021.787745)
Supplement: Supplementary file 1 [file Data_Sheet_1.DOCX]

**Supplementary Table 1. Cox proportional hazard regression analysis with propensity score matching for risks of infectious diseases-related emergency room visits and hospitalizations between subtypes of ADHD and non- ADHD youths stratified by demographic variables and comorbidities**

|  | All emergency visits | | Infectious diseases-related emergency visits | | All hospitalizations | | Infectious diseases-related hospitalizations | |
| --- | --- | --- | --- | --- | --- | --- | --- | --- |
| Variable | aHR (95% CI) | p | aHR (95% CI) | p | aHR (95% CI) | p | aHR (95% CI) | p |
| *Inattention subtype* |  |  |  |  |  |  |  |  |
| ADHD | 1.26 (1.23-1.29) | <.001 | 1.25 (1.20-1.29) | <.001 | 1.28 (1.24-1.31) | <.001 | 1.29 (1.25-1.33) | <.001 |
| Male Sex  (reference = non-ADHD) | 1.27 (1.24-1.30) | <.001 | 1.26 (1.21-1.31) | <.001 | 1.25 (1.22-1.29) | <.001 | 1.28 (1.23-1.32) | <.001 |
| Female sex  (reference = non-ADHD) | 1.25 (1.19-1.30) | <.001 | 1.20 (1.13-1.28) | <.001 | 1.35 (1.28-1.42) | <.001 | 1.32 (1.25-1.41) | <.001 |
| Age ≤ 12  (reference = non-ADHD) | 1.25 (1.21-1.30) | <.001 | 1.21 (1.15-1.27) | <.001 | 1.27 (1.23-1.32) | <.001 | 1.24 (1.18-1.30) | <.001 |
| Age >12  (reference = non-ADHD) | 1.30 (1.26-1.34) | <.001 | 1.29 (1.23-1.35) | <.001 | 1.28 (1.24-1.32) | <.001 | 1.33 (1.28-1.39) | <.001 |
| Autism spectrum disorder (reference = non-ADHD) | 1.05 (0.78-1.42) | 0.740 | 1.16 (0.76-1.77) | 0.498 | 1.07 (0.79-1.46) | 0.650 | 1.19 (0.81-1.75) | 0.376 |
| Tic disorders  (reference = non-ADHD) | 0.90 (0.76-1.06) | 0.190 | 0.97 (0.78-1.22) | 0.806 | 1.10 (0.92-1.31) | 0.315 | 1.03 (0.84-1.27) | 0.78 |
| Epilepsy  (reference = non-ADHD) | 1.21 (1.08-1.36) | <.001 | 1.20 (1.02-1.42) | 0.027 | 0.98 (0.88-1.10) | 0.768 | 0.95 (0.84-1.08) | 0.441 |
| ODD/CD  (reference = non-ADHD) | 1.38 (0.94-2.03) | 0.104 | 0.88 (0.51-1.50) | 0.631 | 1.17 (0.78-1.76) | 0.440 | 1.15 (0.71-1.89) | 0.565 |
| Major depressive disorder (reference = non-ADHD) | 1.01 (0.59-1.72) | 0.969 | 0.85 (0.41-1.75) | 0.654 | 1.04 (0.57-1.88) | 0.906 | 0.86 (0.45-1.66) | 0.660 |
| Intellectual disabilities (reference = non-ADHD) | 0.99 (0.82-1.20) | 0.956 | 0.98 (0.76-1.27) | 0.871 | 0.77 (0.63-0.93) | 0.006 | 0.75 (0.61-0.93) | 0.008 |
| *Hyperactivity subtype* |  |  |  |  |  |  |  |  |
| ADHD | 1.26 (1.24-1.29) | <.001 | 1.23 (1.20-1.26) | <.001 | 1.30 (1.27-1.32) | <.001 | 1.29 (1.26-1.32) | <.001 |
| Male Sex  (reference = non-ADHD) | 1.26 (1.24-1.28) | <.001 | 1.22 (1.18-1.25) | <.001 | 1.28 (1.25-1.31) | <.001 | 1.27 (1.23-1.30) | <.001 |
| Female sex  (reference = non-ADHD) | 1.28 (1.22-1.34) | <.001 | 1.27 (1.19-1.35) | <.001 | 1.38 (1.32-1.45) | <.001 | 1.40 (1.33-1.48) | <.001 |
| Age ≤ 12  (reference = non-ADHD) | 1.26 (1.23-1.29) | <.001 | 1.21 (1.16-1.25) | <.001 | 1.28 (1.25-1.32) | <.001 | 1.27 (1.23-1.32) | <.001 |
| Age >12  (reference = non-ADHD) | 1.30 (1.26-1.33) | <.001 | 1.26 (1.22-1.31) | <.001 | 1.31 (1.27-1.34) | <.001 | 1.30 (1.26-1.34) | <.001 |
| Autism spectrum disorder (reference = non-ADHD) | 1.12 (0.89-1.40) | 0.325 | 1.06 (0.76-1.46) | 0.741 | 0.91 (0.72-1.16) | 0.44 | 0.96 (0.72-1.28) | 0.762 |
| Tic disorders  (reference = non-ADHD) | 1.01 (0.89-1.14) | 0.916 | 1.07 (0.89-1.28) | 0.474 | 1.00 (0.87-1.15) | 1.000 | 1.01 (0.86-1.18) | 0.943 |
| Epilepsy  (reference = non-ADHD) | 1.22 (1.11-1.34) | <.001 | 1.23 (1.07-1.41) | 0.004 | 0.96 (0.87-1.05) | 0.372 | 0.98 (0.89-1.09) | 0.741 |
| ODD/CD  (reference = non-ADHD) | 1.20 (0.92-1.57) | 0.170 | 1.11 (0.74-1.66) | 0.604 | 1.28 (0.96-1.71) | 0.090 | 1.15 (0.83-1.59) | 0.417 |
| Major depressive disorder (reference = non-ADHD) | 1.06 (0.68-1.66) | 0.788 | 1.02 (0.50-2.08) | 0.947 | 1.39 (0.86-2.24) | 0.181 | 0.95 (0.54-1.65) | 0.843 |
| Intellectual disabilities (reference = non-ADHD) | 1.06 (0.91-1.24) | 0.427 | 1.09 (0.88-1.35) | 0.421 | 0.74 (0.63-0.86) | <.001 | 0.81 (0.69-0.96) | 0.017 |

ADHD = attention deficit hyperactivity disorder; CD =conduct disorder; ODD =oppositional defiant disorder

Adjusted cox proportional hazard regression analysis with propensity score matching was conducted adjusted for variable list in Table 1.

Supplementary Table 2. Cox proportional hazard regression model analysis of use of short-acting or long-acting methylphenidate of on hospitalizations and emergency room visits in ADHD youth stratified by demographic variables and comorbidities

|  | All emergency visit | | | Infectious diseases-related emergency visits | | | All hospitalizations | | | Infectious diseases-related hospitalizations | |
| --- | --- | --- | --- | --- | --- | --- | --- | --- | --- | --- | --- |
| Variable | HR (95% CI) | p | HR (95% CI) | | p | HR (95% CI) | | p | HR (95% CI) | | p |
| Use of short-acting methylphenidate  (reference = non-users) ^a^ | 0.21 (0.20-0.22) | <.001 | 0.38 (0.36-0.41) | | <.001 | 0.93 (0.91-0.96) | | <.001 | 0.80 (0.77-0.83) | | <.001 |
| Male sex  (reference = non-users) | 0.21 (0.20-0.22) | <.001 | 0.39 (0.36-0.41) | | <.001 | 0.94 (0.91-0.97) | | <.001 | 0.82 (0.79-0.86) | | <.001 |
| Female sex  (reference = non-users) | 0.20 (0.18-0.23) | <.001 | 0.38 (0.33-0.43) | | <.001 | 0.91 (0.86-0.97) | | 0.003 | 0.73 (0.67-0.79) | | <.001 |
| Younger age  (reference = non-users) | 0.14 (0.13-0.16) | <.001 | 0.32 (0.29-0.36) | | <.001 | 0.89 (0.84-0.93) | | <.001 | 0.63 (0.59-0.67) | | <.001 |
| Older age  (reference = non-users) | 0.24 (0.23-0.25) | <.001 | 0.41 (0.39-0.44) | | <.001 | 0.95 (0.92-0.98) | | 0.004 | 0.86 (0.82-0.90) | | <.001 |
| Autism spectrum disorder (reference = non-users) | 0.26 (0.23-0.29) | <.001 | 0.39 (0.34-0.46) | | <.001 | 0.89 (0.82-0.96) | | 0.003 | 0.72 (0.65-0.81) | | <.001 |
| Tic disorders  (reference = non-users) | 0.23 (0.20-0.27) | <.001 | 0.38 (0.31-0.47) | | <.001 | 1.09 (0.97-1.22) | | 0.13 | 0.93 (0.79-1.09) | | 0.356 |
| Epilepsy  (reference = non-users) | 0.29 (0.25-0.34) | <.001 | 0.46 (0.37-0.56) | | <.001 | 0.96 (0.87-1.07) | | 0.471 | 0.91 (0.78-1.06) | | 0.221 |
| ODD/CD  (reference = non-users) | 0.21 (0.19-0.24) | <.001 | 0.35 (0.29-0.41) | | <.001 | 1.09 (0.99-1.20) | | 0.079 | 0.90 (0.79-1.02) | | 0.107 |
| Major depressive disorder (reference = non-users) | 0.27 (0.22-0.34) | <.001 | 0.46 (0.34-0.62) | | <.001 | 0.91 (0.75-1.11) | | 0.372 | 0.75 (0.58-0.98) | | 0.034 |
| Intellectual disabilities (reference = non-users) | 0.28 (0.25-0.31) | <.001 | 0.41 (0.36-0.47) | | <.001 | 0.91 (0.85-0.97) | | 0.005 | 0.82 (0.75-0.90) | | <.001 |
| Use of long-acting methylphenidate  (reference = non-users) ^a^ | 0.09 (0.09-0.10) | <.001 | 0.28 (0.26-0.29) | | <.001 | 0.97 (0.94-1.00) | | 0.080 | 0.86 (0.82-0.89) | | <.001 |
| Male sex  (reference = non-users) | 0.09 (0.09-0.10) | <.001 | 0.28 (0.26-0.30) | | <.001 | 0.98 (0.95-1.01) | | 0.168 | 0.86 (0.82-0.90) | | <.001 |
| Female sex  (reference = non-users) | 0.10 (0.09-0.11) | <.001 | 0.26 (0.23-0.30) | | <.001 | 0.95 (0.88-1.02) | | 0.132 | 0.83 (0.76-0.92) | | <.001 |
| Younger age  (reference = non-users) | 0.06 (0.05-0.06) | <.001 | 0.22 (0.20-0.25) | | <.001 | 0.96 (0.91-1.02) | | 0.207 | 0.71 (0.66-0.77) | | <.001 |
| Older age  (reference = non-users) | 0.11 (0.10-0.11) | <.001 | 0.30 (0.28-0.32) | | <.001 | 0.98 (0.94-1.01) | | 0.214 | 0.91 (0.87-0.96) | | <.001 |
| Autism spectrum disorder (reference = non-users) | 0.13 (0.12-0.15) | <.001 | 0.29 (0.25-0.34) | | <.001 | 0.98 (0.90-1.07) | | 0.635 | 0.74 (0.65-0.83) | | <.001 |
| Tic disorders  (reference = non-users) | 0.11 (0.09-0.12) | <.001 | 0.29 (0.23-0.35) | | <.001 | 0.95 (0.84-1.07) | | 0.371 | 0.87 (0.73-1.03) | | 0.098 |
| Epilepsy  (reference = non-users) | 0.12 (0.10-0.14) | <.001 | 0.31 (0.25-0.39) | | <.001 | 1.01 (0.90-1.13) | | 0.908 | 0.97 (0.82-1.15) | | 0.731 |
| ODD/CD  (reference = non-users) | 0.09 (0.08-0.11) | <.001 | 0.27 (0.23-0.32) | | <.001 | 0.96 (0.87-1.05) | | 0.386 | 0.95 (0.83-1.09) | | 0.479 |
| Major depressive disorder (reference = non-users) | 0.13 (0.10-0.16) | <.001 | 0.31 (0.23-0.42) | | <.001 | 0.94 (0.77-1.15) | | 0.552 | 0.89 (0.68-1.17) | | 0.407 |
| Intellectual disabilities (reference = non-users) | 0.12 (0.11-0.14) | <.001 | 0.29 (0.26-0.33) | | <.001 | 0.98 (0.91-1.05) | | 0.561 | 0.85 (0.77-0.95) | | 0.002 |

CD =conduct disorder; ODD =oppositional defiant disorder.
There were 28,377 users of short-acting methylphenidate, 20,013 users of long-acting methylphenidate and non-users of methylphenidate.

This analysis was conducted using the propensity score matching the sample of ADHD between methylphenidate and non-methylphenidate in Table 3.

Supplementary Table 3. Conditional Poisson regression model for self-controlled case series study design of hospitalizations and emergency room visits in ADHD youths with use of methylphenidate and emergency room visit

|  | All emergency visit | | | Infectious diseases-related emergency visits | | | All hospitalizations | | | Infectious diseases-related hospitalizations | | |
| --- | --- | --- | --- | --- | --- | --- | --- | --- | --- | --- | --- | --- |
| Variable | RR^a^ | 95% CI | p | RR | 95% CI | p | RR^a^ | 95% CI | p | RR^a^ | 95% CI | p |
|  | n=15501 | | | n=7790 | | | n=18471 | | | n=12187 | | |
| Exposures of short-acting methylphenidate | | | |  |  |  |  |  |  |  |  |  |
| 0~30 days | 0.74 | (0.67-0.82) | <0.001 | 0.84 | (0.73-0.95) | 0.007 | 0.22 | (0.19-0.26) | <0.001 | 0.19 | (0.15-0.23) | <0.001 |
| 30~60 days | 0.74 | (0.65-0.84) | <0.001 | 0.94 | (0.81-1.10) | 0.460 | 0.19 | (0.15-0.24) | <0.001 | 0.18 | (0.14-0.24) | <0.001 |
| 60~90 days | 0.80 | (0.69-0.92) | <0.001 | 0.93 | (0.77-1.11) | 0.415 | 0.22 | (0.17-0.28) | <0.001 | 0.17 | (0.12-0.24) | <0.001 |
| 0~90 days | 0.75 | (0.70-0.81) | <0.001 | 0.89 | (0.81-0.97) | 0.018 | 0.21 | (0.19-0.24) | <0.001 | 0.18 | (0.16-0.21) | <0.001 |
|  | n=11287 | | | n=5541 | | | n=13073 | | | n=8577 | | |
| Exposures of long-acting methylphenidate | | | |  |  |  |  |  |  |  |  |  |
| 0~30 days | 0.49 | (0.43-0.56) | <0.001 | 0.76 | (0.66-0.89) | <0.001 | 0.20 | (0.17-0.24) | <0.001 | 0.20 | (0.16-0.26) | <0.001 |
| 30~60 days | 0.60 | (0.51-0.70) | <0.001 | 0.68 | (0.56-0.84) | <0.001 | 0.20 | (0.15-0.25) | <0.001 | 0.15 | (0.10-0.21) | <0.001 |
| 60~90 days | 0.57 | (0.46-0.69) | <0.001 | 0.68 | (0.53-0.87) | <0.001 | 0.19 | (0.13-0.26) | <0.001 | 0.17 | (0.11-0.26) | <0.001 |
| 0~90 days | 0.54 | (0.49-0.59) | <0.001 | 0.72 | (0.65-0.81) | <0.001 | 0.20 | (0.17-0.23) | <0.001 | 0.18 | (0.15-0.22) | <0.001 |

^a^Relative incidence (RR) was calculated by conditional Poisson regression, adjusted for all time-invariant covariates that are constant within each individual during the follow-up and time-varying covariate (i.e., age stage).
